# Podocyte Glucocorticoid Receptors Are Essential for Glomerular Endothelial Cell Homeostasis in Diabetes Mellitus

**DOI:** 10.1161/JAHA.120.019437

**Published:** 2021-07-26

**Authors:** Swayam Prakash Srivastava, Han Zhou, Ocean Setia, Alan Dardik, Carlos Fernandez‐Hernando, Julie Goodwin

**Affiliations:** ^1^ Department of Pediatrics Yale University School of Medicine New Haven CT; ^2^ Vascular Biology and Therapeutics Program Yale University School of Medicine New Haven CT; ^3^ Department of Surgery Yale University School of Medicine New Haven CT; ^4^ Department of Surgery VA Connecticut Healthcare Systems West Haven CT; ^5^ Department of Comparative Medicine Yale University School of Medicine New Haven CT; ^6^ Program in Integrative Cell Signaling and Neurobiology of Metabolism (ICSNM) Yale University School of Medicine New Haven CT; ^7^ Department of Pathology Yale University School of Medicine New Haven CT

**Keywords:** diabetes mellitus, endothelium, glucocorticoid receptor, podocyte, Wnt signaling, Animal Models of Human Disease, Fibrosis, Nephrology and Kidney

## Abstract

**Background:**

Proteinuria and glomerular segmental fibrosis are inevitable complications of diabetic nephropathy though their mechanisms are poorly understood. Understanding the clinical characteristics and pathogenesis of proteinuria and glomerular segmental fibrosis in diabetic nephropathy is, therefore, urgently needed for patient management of this severe disease.

**Methods and Results:**

Diabetes mellitus was induced in podocyte‐specific glucocorticoid receptor knockout (GR^PKO^) mice and control littermates by administration of streptozotocin. Primary podocytes were isolated and subjected to analysis of Wnt signaling and fatty acid metabolism. Conditioned media from primary podocytes was transferred to glomerular endothelial cells. Histologic analysis of kidneys from diabetic GR^PKO^ mice showed worsened fibrosis, increased collagen deposition, and glomerulomegaly indicating severe glomerular fibrosis. Higher expression of transforming growth factor‐βR1 and β‐catenin and suppressed expression of carnitine palmitoyltransferase 1A in nephrin‐positive cells were found in the kidneys of diabetic GR^PKO^ mice. Podocytes isolated from diabetic GR^PKO^ mice demonstrated significantly higher profibrotic gene expression and suppressed fatty acid oxidation compared with controls. Administration of a Wnt inhibitor significantly improved the fibrotic features in GR^PKO^ mice. The glomerular endothelium of diabetic GR^PKO^ mice demonstrated the features of endothelial‐to‐mesenchymal transition. Moreover, endothelial cells treated with conditioned media from podocytes lacking GR showed increased expression of α‐smooth muscle actin, transforming growth factor‐βR1 and β‐catenin levels.

**Conclusions:**

These data demonstrate that loss of podocyte GR leads to upregulation of Wnt signaling and disruption in fatty acid metabolism. Podocyte–endothelial cell crosstalk, mediated through GR, is important for glomerular homeostasis, and its disruption likely contributes to diabetic nephropathy.

**Nonstandard Abbreviations and Acronyms**
ACR: albumin‐to‐creatinine ratio
DN: diabetic nephropathy
EndMT: endothelial‐to‐mesenchymal transition
FAO: fatty acid oxidation
GR: glucocorticoid receptor
GR^PKO^
: podocyte‐specific glucocorticoid receptor knockout
TGF: transforming growth factor
UUO: unilateral ureteral obstruction

**Clinical Perspective**

**What Is New?**

- This study demonstrates that deficiency of the podocyte glucocorticoid receptor negatively impacts glomerular endothelial cell health in diabetes mellitus by upregulation of both Wnt signaling and profibrotic transition programs and suppression of fatty acid oxidation.
- Results highlight the crucial nature of endogenous steroid signaling in podocytes in influencing the diabetic nephropathy phenotype.

**What Are the Clinical Implications?**

- Podocyte‐specific steroid delivery technologies may represent a novel therapeutic target for modulation of central metabolism and fibrogenic processes in diabetic nephropathy.

Proteinuria and diabetic nephropathy (DN) are inevitable complications of long‐standing diabetes mellitus, both types I and II, though their mechanisms are poorly understood. Diabetic nephropathy has no known cure and is a major source of end‐stage renal disease worldwide. Pathologically, the key histologic features of DN are podocyte loss, mesangial expansion, increased glomerulosclerosis and interstitial fibrosis, and thickening of the glomerular basement membrane.^1^, ^2^ Glomerular fibrosis is one key feature of DN that is poorly understood. It is characterized by excess deposition of extracellular matrix, loss of capillary networks, accumulation of fibrillary collagens, activated myofibroblasts, and inflammatory cells.^3^

Current standards of care, which include angiotensin‐converting enzyme inhibitors, angiotensin II receptor blockers, and statins, provide reduction of proteinuria only by hemodynamic perturbations and do not address the underlying factors that incite and perpetuate DN.^4^, ^5^, ^6^, ^7^, ^8^ A number of renoprotective agents, including sodium glucose transporter‐2 inhibitors, dipeptidyl peptidase‐4 inhibitors and statins, have been studied in mouse models and controlled clinical trials,^9^, ^10^, ^11^, ^12^, ^13^ and indeed there have been some promising advancements in this regard in recent years. For example, a randomized clinical trial of the sodium glucose transporter‐2 inhibitor canagliflozin demonstrated a lower risk of kidney failure and cardiovascular events compared with the placebo group after a mean follow‐up time of about 2.5 years,^14^ and similar protective results were shown in a randomized clinical trial of dapagliflozin in patients with chronic kidney disease, both with and without diabetes mellitus.^15^ Additionally, a large randomized clinical trial of the selective mineralocorticoid receptor antagonist finerenone was shown to result in a lower risk of both chronic kidney disease progression and cardiovascular events in patients with type 2 diabetes mellitus, compared with placebo. In the SONAR (Study of Diabetic Nephropathy With Atrasentan) study, a large, double‐blind, placebo‐controlled trial, the selective endothelin A antagonist atrasentan was shown to be renoprotective in patients with type 2 diabetes mellitus and chronic kidney disease stages 2 to 4.^16^, ^17^ Though these developments are encouraging, the complicated pathogenesis of DN, which involves multiple cell types, signaling molecules and pathways, and metabolic factors, continues to stimulate investigation of additional novel therapeutics.

Recent data indicate that the canonical Wnt/β‐catenin signaling pathway is an important mediator of renal disease, including proteinuric diseases such as diabetes mellitus.^18^, ^19^, ^20^, ^21^ The Wnt gene family is composed of 19 genes that are structurally related and act as extracellular signaling factors.^22^ There are 2 major subdivisions of the Wnt pathway: the canonical arm, which results in activation of β‐catenin, and the noncanonical pathway, which can be subdivided into the planar cell polarity pathway and a calcium‐dependent pathway. It is well known that Wnt signaling plays a key role in development, and in particular vascular development, of the central nervous system, eye, and reproductive system.^23^ The planar cell polarity arm of Wnt signaling plays a role in proper cellular patterning. Some noncanonical Wnt ligands, including Wnt9b and Wnt11, have been found to be particularly important during kidney morphogenesis, as they participate in development of mesonephric and metanephric tubules as well as ureteric branching.^24^

Both canonical and noncanonical Wnt signaling seem to play a role in the progression of chronic kidney disease, with the noncanonical effects considerably less well understood.^25^ In other disease states, including cardiovascular disease and nonalcoholic fatty liver disease, the noncanonical Wnt ligand Wnt5a is implicated mechanistically via its stimulation of c‐Jun N‐terminal kinase and induction of endothelial dysfunction.^25^

Recently, there is recognition that the Wnt signaling pathway plays a key role in the progression of DN and specifically that this pathway is upregulated under diabetic conditions.^26^ For example, Wnt proteins have been shown to be upregulated in the kidneys of both type 1 and 2 diabetic animal models.^26^ In addition, high glucose can activate Wnt signaling in human renal proximal tubular epithelial cells, and this effect can be blocked by insulin.^26^ There is evidence that the Wnt signaling pathway has specific effects on mesangial cells,^27^ renal tubular cells,^28^ and podocytes.^29^, ^30^, ^31^ Furthermore, podocyte‐specific overexpression of β‐catenin in mice results in glomerular basement membrane damage and albuminuria suggesting that β‐catenin is a key contributor that can disrupt glomerular basement membrane integrity and function.^31^ However, since activation of β‐catenin is the final step in this very complex signaling pathway, the molecular mechanisms by which the Wnt pathway contributes to DN and podocyte damage remain poorly understood.

Glucocorticoids and the glucocorticoid receptor (GR) play an important role in lipid homeostasis in multiple microenvironments including adipose, liver, and peripheral tissues; however, excess glucocorticoid/GR signaling can disrupt adipocyte differentiation and cellular metabolism.^32^ Dysregulated fatty acid metabolism and lipotoxicity in podocytes are critically associated with loss of podocyte function and podocyte injury, and contribute to extracellular matrix deposition.^32^ Defective central metabolism, which is characterized by suppressed fatty acid oxidation (FAO) and abnormal glucose metabolism are key events in mesenchymal activation and myofibroblast formation in diabetic kidneys.^8^, ^33^, ^34^ We have recently shown that loss of the endothelial GR augments Wnt signaling, suppresses FAO and activates mesenchymal transition in diabetic kidneys, which results in acceleration of DN.^35^ Podocytes are highly susceptible to free fatty acid, and are dependent on FAO for their function.^32^ Therefore, defective fatty acid metabolism in podocytes may to contribute to proteinuria and glomerular fibrosis in diabetic kidneys.

We previously developed a mouse model with tissue‐specific podocyte glucocorticoid receptor knockout (GR^PKO^) and demonstrated that this receptor is critical in limiting proteinuria in settings of renal injury.^36^ Here, we demonstrate that this injury is mediated, in part, by upregulation of transforming growth factor (TGF)β signaling and Wnt signaling and suppression of FAO in podocytes lacking GR. The cumulative effect of these metabolic derangements leads to a profibrotic phenotype in endothelial cells and eventually glomerular fibrosis.

## METHODS

The data that support the findings of this study are available from the corresponding author upon reasonable request.

### Antibodies and Reagents

Rabbit polyclonal anti‐GR (Cat: SAB4501309) and mouse monoclonal anti‐α‐smooth muscle actin (Cat: A5228) antibodies were purchased from Sigma (St Louis, MO). Anti–TGFβR1 (Cat: ab31013), anti‐phospho‐smad2 (ab53100), and rabbit polyclonal collagen I (ab34710) were obtained from Abcam (Cambridge, UK). Mouse anti‐β‐catenin (Cat: 610154) and rat anti‐CD31 antibodies (Cat: 550274) were from BD Biosciences (Franklin Lakes, NJ). Fluorescence‐, Alexa Fluor 647–, and rhodamine‐conjugated secondary antibodies were obtained from Jackson ImmunoResearch (West Grove, PA). Reagents included streptozotocin and etomoxir (Sigma).

### Animal Experiments

Male podocyte GR^PKO^ mice and control littermates, aged 8 to 12 weeks, were bred as previously described and used in these studies.^36^ Given the worsened DN phenotype observed in males, compared with females, only male mice were used in these studies. All experiments were performed according to a protocol approved by the Institutional Animal Care and Use Committee at the Yale University School of Medicine and were in accordance with the National Institutes of Health *Guidelines for the Care of Laboratory Animals*. Briefly, diabetes mellitus was induced in GR^PKO^ mice with 5 consecutive intraperitoneal doses of streptozotocin 50 mg/kg in 10 mmol/L citrate buffer (pH 4.5),^8^, ^37^ and mice were monitored over a period of 16 weeks. A Wnt inhibitor (LGK974) was provided via gavage to GR^PKO^ and control littermates at a dose of 5 mg/kg per day, 6 days per week for 8 weeks,^38^ and was started 16 weeks after streptozotocin injections. Etomoxir (20 mg/kg), a carnitine palmitoyltransferase 1A inhibitor, was administered intraperitoneally 3 times a week for 3 weeks; this dosing regimen is similar to previously published studies examining fatty acid metabolism inhibition in rodents.^39^, ^40^ After euthanasia, blood and tissues were harvested. Postprandial blood glucose was measured by using a Contour Ultra blood glucose meter.

### Mouse Model of Unilateral Ureteral Obstruction

Unilateral ureteral obstruction (UUO) surgery procedure was performed as previously described.^41^ Briefly, mice were anesthetized with isoflurane (3%–5% for induction and 1%–3% for maintenance). Mice were shaved on the left side of the abdomen, a vertical incision was made through the skin with a scalpel, and the skin was retracted. A second incision was made through the peritoneum to expose the kidney. The left ureter was tied twice 15 mm below the renal pelvis with surgical silk, and the ureter was then severed between the 2 ligatures. Then, the kidney was placed gently back into its correct anatomic position, and sterile saline was added to replenish fluid loss. The incisions were sutured and mice were individually caged. Buprenorphine was used as an analgesic. A first dose was administered 30 minutes before surgery and then every 12 hours for 72 hours, at a dose of 0.05 mg/kg subcutaneously. Mice were sacrificed and kidneys were harvested after perfusion with PBS at 12 days after UUO. Contralateral kidneys were used as a nonfibrotic control for all experiments using this model.

### Serum and Urine Assays

Spot urine samples were collected from first morning urines of spontaneously voiding mice. Urine albumin levels were estimated using a Mouse Albumin ELISA Kit (Exocell, Philadelphia, PA). Blood glucose was measured by using a Contour Ultra blood glucose meter.

### Isolation of Endothelial Cells

Endothelial cells from the kidneys of nondiabetic and diabetic mice were isolated using a standardized kit (Miltenyi Biotec, Auburn, CA) by following the manufacturer's instructions. Briefly, kidneys were isolated and minced into small pieces. Using a series of enzymatic reactions by treating the tissue with trypsin and collagenase type I solution, a single cell suspension was created. The pellet was dissolved with CD31 magnetic beads, and the CD31‐labeled cells were separated on a magnetic separator. The cells were further purified on a column. Cell number was counted by hemocytometer and cells were plated on 0.1% gelatin‐coated Petri dishes.

### Kidney Histology

Sirius red, periodic acid‐Schiff, and Masson‐Trichrome staining were performed by the Yale Research Histology Core and visualized using an Aperio imaging system. Masson‐Trichrome–stained sections were evaluated by ImageJ software, and the fibrotic areas were estimated. For Sirius red staining, deparaffinized sections were incubated with picrosirius red solution for 1 hour at room temperature. The slides were washed twice with acetic acid solution for 30 seconds per wash. Then, the slides were dehydrated in absolute alcohol 3 times. The slides were cleared in xylene and mounted with a synthetic resin. For each mouse, images of 6 different fields of view were evaluated at ×40 magnification, and 15 to 20 stained glomeruli from each mouse were analyzed. Masson‐Trichrome stain relative area of fibrosis and Sirius red relative collagen deposition were analyzed to calculate interstitial fibrosis and collagen deposition, respectively. Glomerular surface area was calculated using traced glomeruli and ImageJ algorithms, and mesangial area was calculated by ImageJ using a point counting method according to previously published studies.^42^

### Immunofluorescence

Frozen kidney sections (5 μm) were used for immunofluorescence; double‐positive labeling with TGFβR1/nephrin, β‐catenin/nephrin, CPT1a/nephrin, αSMA/CD31, TGFβR1/CD31 and β‐catenin/CD31 were measured. Briefly, sections were fixed in 100% ethyl alcohol followed by rehydration in PBS. They were then permeabilized with 0.2% Triton and blocked with a solution of 10% serum and 1% BSA. Thereafter, the sections were incubated with the primary antibodies (1:100) overnight and washed in PBS (5 minutes) 3 times. Sections were washed, and secondary antibodies were incubated for 45 minutes in the dark. Sections were washed again, and coverslips were mounted using Vectashield. Sections were analyzed and quantified by ImageJ. For each mouse, original magnification of Å ≈ 400 pictures was obtained from 6 different areas, and quantification was performed. We analyzed 15 to 20 glomeruli from each mouse.

### Immunohistochemistry

Kidney sections (5 μm thick) were used for immunohistochemistry. To block endogenous peroxidase, all sections were incubated in 0.3% hydrogen peroxide for 10 minutes. Immunohistochemical staining was performed using a Vectastain ABC Kit (Vector Laboratories, Burlingame, CA). Rabbit polyclonal TGFβR1 (1:100) and rabbit polyclonal p‐smad2 (1:100) antibodies were purchased from Abcam (Cambridge, MA). Mouse anti–β‐catenin antibody (1:100) was purchased from BD Biosciences. In the negative controls, the primary antibody was omitted and replaced with the blocking solution. We analyzed 15 to 20 stained glomeruli from each mouse.

### Isolation of Primary Podocytes

Podocyte isolation was performed as previously described.^36^ Briefly, kidneys were dissected, minced, and digested for 45 to 60 minutes in a solution of collagenase A (1 mg/mL) (Roche) and DNAse I. The resulting suspension was strained through a 100‐µm strainer and washed 3 times with Hanks Balanced Salt Solution buffer. Then, the suspension was resuspended in 30 mL of a 45% Percoll solution (GE‐Healthcare BioSciences) in isotonic buffer and centrifuged at 10 000 *g* for 60 minutes at 4°C. Glomeruli were enriched in the top band after centrifugation, and this band was collected. Cells were washed 3 times with Hanks Balanced Salt Solution to remove the Percoll solution. The pellet was resuspended and plated on collagen type I–coated dishes in RPMI 1640 medium with 9% FBS, 100 U/mL penicillin, 100 µg/mL streptomycin, 100 mmol/L HEPES, 1 mmol/L sodium bicarbonate, and 1 mmol/L sodium pyruvate.

### Fatty Acid Uptake

Cultured isolated podocytes were incubated with medium containing 2 μCi [^14^C]‐palmitate for 2 hours. [^14^C]‐palmitate uptake was measured by liquid scintillation counting.

### Fatty Acid Oxidation

Cultured isolated podocytes were incubated with medium containing 0.75 mmol/L palmitate (conjugated to 2% free fatty acid–free BSA/[^14^C] palmitate at 2 μCi/mL) for 2 hours. One milliliter of the culture medium was transferred to a sealable tube, the cap of which housed a Whatman filter paper disc. ^14^CO_2_ trapped in the media was then released by acidification of media using 60% perchloric acid. Radioactivity that had become adsorbed onto the filter discs was then quantified by liquid scintillation counting.

### Quantitative Polymerase Chain Reaction

Total RNA was isolated using standard Trizol protocol. RNA was reverse transcribed using the iScript cDNA Synthesis kit (Bio‐Rad) and quantitative polymerase chain reaction was performed on a Bio‐Rad C1000 Touch thermal cycler using the resultant cDNA, quantitative polymerase chain reaction master mix, and gene‐specific primers. The following primers were used:

α‐SMA: Forward 5′‐CTGACAGAGGCACCACTGAA and Reverse 5′‐GAAATAGCCAAGCTCAG
CD31: Forward 5′‐AGGCTTGCATAGAGCTCCAG and Reverse 5′‐TTCTTGGTTTCCAGCTATGG
FSP‐1: Forward 5′‐TTCCAGAAGGTGATGAG and Reverse 5′‐TCATGGCAATGCAGGACAGGAAGA
TGFβR1: Forward 5′CGTGTGCCAAATGAAGAGGAT and Reverse 5′‐AAGGTGGTGCCCTCTGAAATG
Axin2: Forward 5′ AACCTATGCCCGTTTCCTCTA and Reverse 5′‐GAGTGTAAAGACTTGGTCCACC
β‐catenin: Forward 5′‐TGACACCTCCCAAGTCCTTT and Reverse 5′ TTGCATACTGCCCGTCAAT
PGC1a: Forward 5'‐AGTCCCATACACAACCGCAG and Reverse5'‐CCCTTGGGGTCATTTGGTGA
Collagen 1: Forward 5'‐ATCTCCTGGTGCTGATGGAC and Reverse 5'‐ ACCTTGTTTGCCAGGTTCAC
Fibronectin: Forward 5'‐CGAGGTGACAGAGACCACAA and Reverse 5'‐CTGGAGTCAAGCCAGACACA
IL‐1β: Forward 5'‐CCAAGCAACGACAAAATACC and Reverse 5'‐GTTGAAGACAAACCGTTTTTCC
IL‐6: Forward 5′‐TCTGAAGGACTCTGGCTTTG and Reverse 5′‐GATGGATGCTACCAAACTGGA
Cpt1a: Forward 5′‐ GGTCTTCTCGGGTCGAAAGC and Reverse 5′‐ TCCTCCCACCAGTCACTCAC
18S: Forward 5'‐TTCCGATAACGAACGAGACTCT and Reverse 5'‐ GGCTGAACGCCACTTGTC

Gene expression was normalized to the housekeeping gene 18S and is presented as fold change. All primers were synthesized by the Keck Oligo Synthesis facility at Yale School of Medicine.

### Statistical Analysis

**Figure 1:**
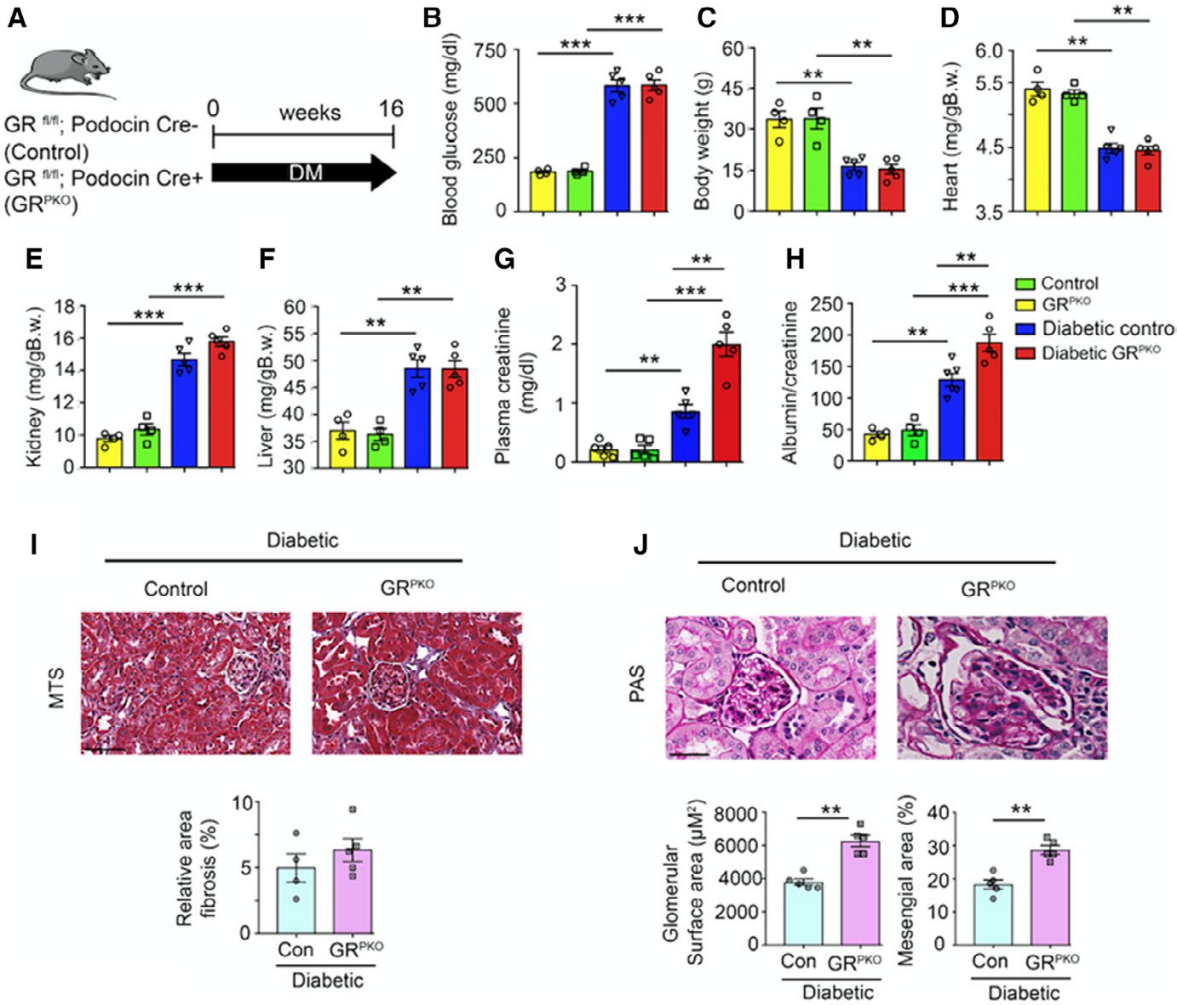
Physiological characteristics of nondiabetic and diabetic podocyte‐specific glucocorticoid receptor knockout (GR^PKO^) mice. **A**, Schematic diagram, showing induction of diabetes mellitus in *GR^fl/fl^; Podocin Cre‐* (control), and *GR^fl/fl^; Podocin Cre+* (GR^PKO^) mice. Five doses of streptozotocin 50 mg/kg per day intraperitoneally were injected to induce fibrosis. After 16 weeks, mice were sacrificed. Physiological parameters: **B,** blood glucose; **C**, body weight; **D**, heart weight/body weight; **E**, kidney weight/body weight; **F**, liver weight/body weight; **G**, plasma creatinine, and **H**, albumin‐to‐creatinine ratio were measured. n=4 to 6 mice/group. **I**, Masson‐Trichrome stain (MTS); **J**, Periodic acid‐Schiff (PAS) staining in kidneys of diabetic control and GR^PKO^ mice were analyzed. Representative images are shown. Relative area fibrosis (%), glomerular surface area (μm^2^), and mesangial area (%) were measured using ImageJ. n=4 to 5 mice/group. Scale bar: 50 μm in each panel. *P<0.05, **P<0.01, ***P<0.001. Data are mean±SEM. Two‐way ANOVA was used to analyze **B** through **H** and nonparametric Mann‐Whitney *U* test was used to analyze **I** and **J**. DM indicates diabetes mellitus.

**Figure 2:**
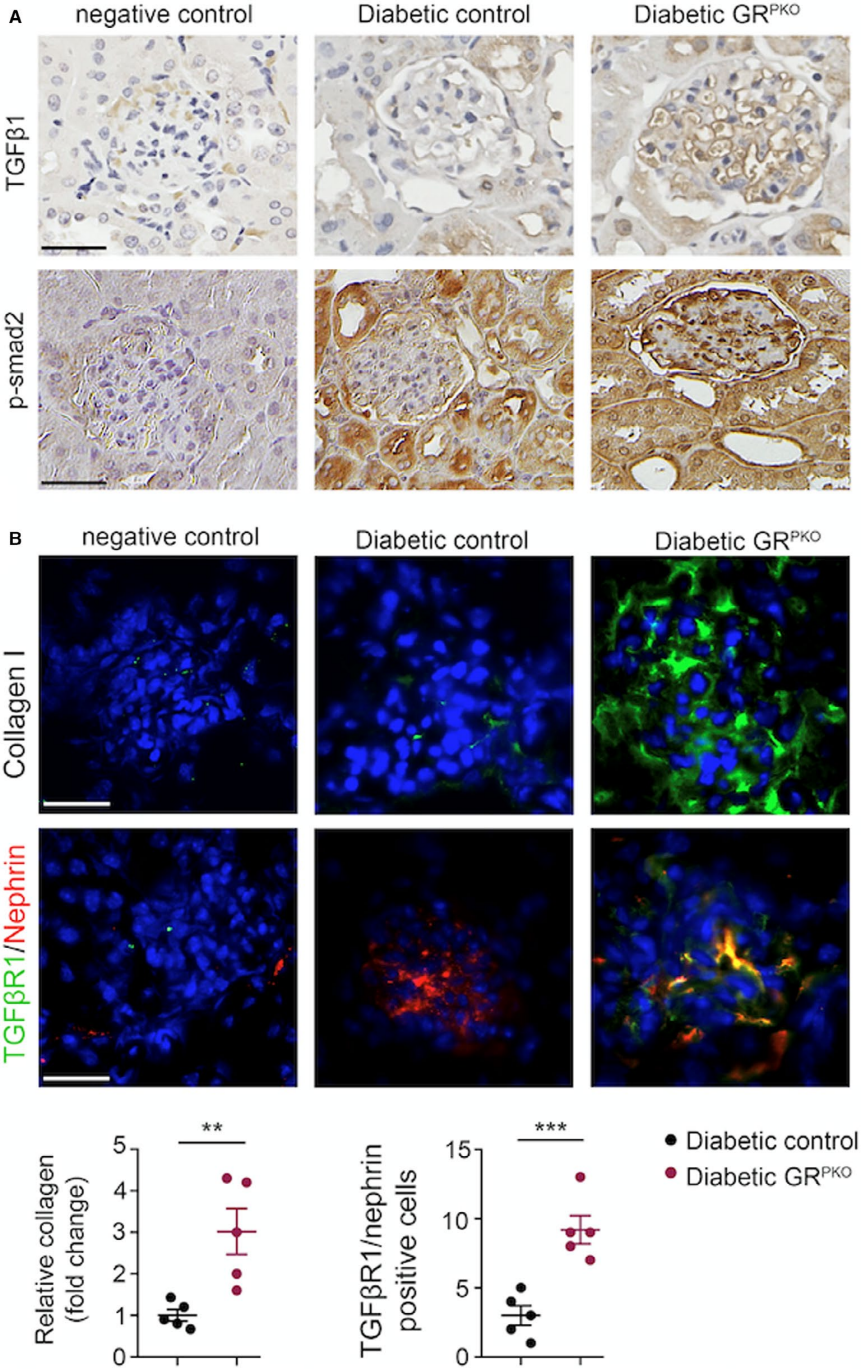
Immunohistochemistry and immunofluorescence. **A**, Immunohistochemistry of transforming growth factor (TGF) β1and p‐smad2 in the glomeruli of diabetic control and podocyte‐specific glucocorticoid receptor knockout (GR^PKO^) mice (n=5/group). Representative pictures are shown. Scale bar 50 μm. **B**, Immunofluorescence analysis of collagen I (fluorescein isothiocyanate [FITC]‐labeled and DAPI blue nuclei, pixel/field) and TGFβR1and nephrin colabeling (FITC‐labeled TGFβR1, rhodamine‐labeled nephrin and DAPI blue nuclei) in the glomeruli of diabetic unilateral ureteral obstruction control and GR^PKO^ mice. Representative images are shown. Scale bar 50 μm in each panel. The number of double‐positive–labeled cells is quantified (n=5/group). **P<0.01; ***P<0.001. Data are mean±SEM. Nonparametric Mann‐Whitney *U* test was used.

**Figure 3:**
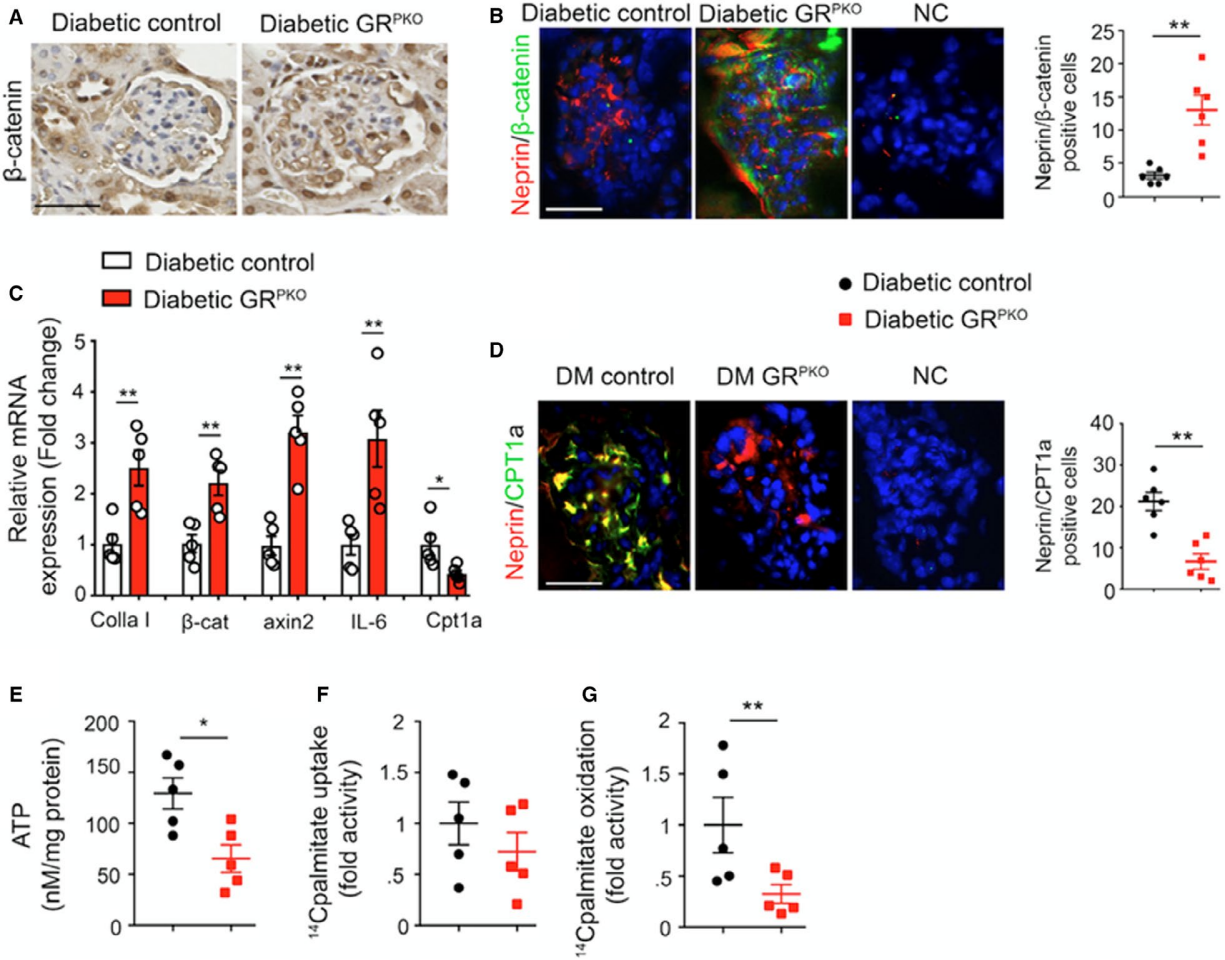
Induction of canonical Wnt signaling is responsible for podocyte glucocorticoid receptor loss‐linked fibrosis in glomeruli of diabetic mice. **A**, Immunohistochemical analysis of β‐catenin in the glomeruli of diabetic control and podocyte‐specific glucocorticoid receptor knockout (GR^PKO^) mice (n=5/group). Representative images are shown. Scale bar 50 μm. **B**, Immunofluorescence analysis of nephrin/β‐catenin co‐labeling in the glomeruli of diabetic control and GR^PKO^ mice (fluorescein isothiocyanate [FITC]‐labeled β‐catenin, rhodamine‐labeled nephrin and DAPI blue nuclei). Representative images are shown. Scale bar 50 μm in each panel (n=5/group). **C**, Quantitative polymerase chain reaction gene expression analysis of *colla1*, *β‐catenin*, *axin2*, *IL‐1β*, *IL‐6,* and *cpt1a* in isolated podocyte‐enriched cultures from the glomeruli of diabetic control and GR^PKO^ mice (n=5/group). **D**, Immunofluorescence analysis of nephrin/CPT1a co‐labeling in the glomeruli of diabetic control and GR^PKO^ mice (FITC‐labeled CPT1a, rhodamine‐labeled nephrin and DAPI blue nuclei). Representative images are shown. Scale bar 50 μm in each panel (n=5/group). **E**, Cellular ATP measurements in isolated podocytes from kidneys of diabetic control and diabetic GR^PKO^ mice (n=5/group). **F**, Radiolabeled [C^14^]palmitate uptake analysis in isolated podocytes from kidneys of diabetic control and diabetic GR^PKO^ mice. CPM of each sample was counted. **G**, Radiolabeled [^14^C]palmitate oxidation and [^14^CO_2_] release were measured. CPM of each sample was counted (n=5/group). Data are shown as mean±SEM. *P<0.05; **P<0.01. Nonparametric Mann‐Whitney *U* Test was used. CPM indicates counts per minute; DM, diabetes mellitus; and NC, negative control.

**Figure 4:**
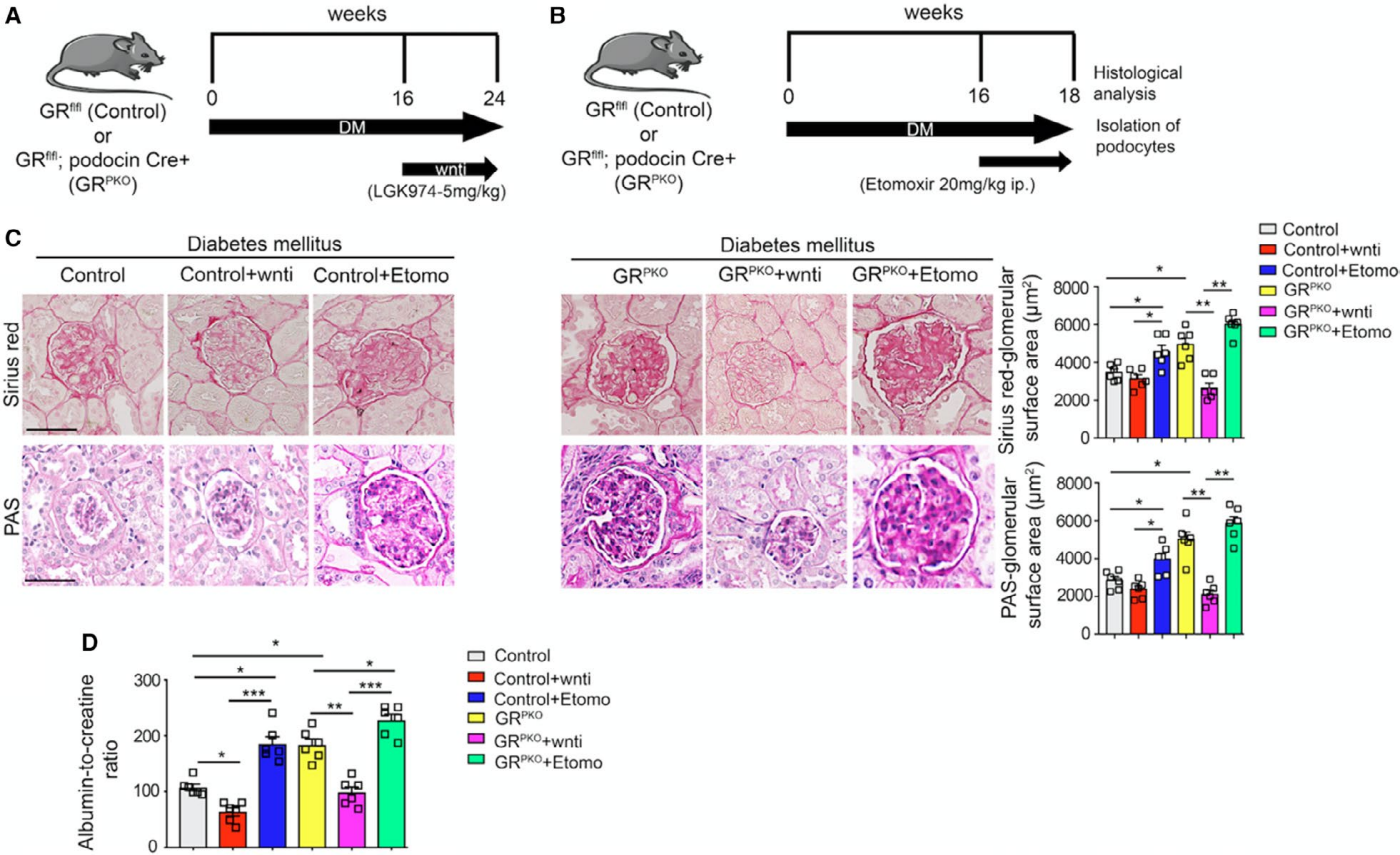
Wnt inhibitor rescues glomerular fibrosis in diabetic control but not podocyte‐specific glucocorticoid receptor knockout (GR^PKO^) mice. **A**, Schematic diagram, showing the treatment protocol of Wnti (LGK974) and, (**B**) etomoxir (CPT1a inhibitor) in diabetic control and GR^PKO^ mice. **C**, Sirius red and Periodic acid‐Schiff staining in the glomeruli of diabetic mice was analyzed. Representative images are shown. Glomerular surface area was measured using ImageJ (n=5 to 6/group; scale bar 50 μm in each panel). **D**, Albumin‐to‐creatinine ratios from mice of each genotype subjected to either LGK974 or etomoxir (N=6 mice/group). Data are mean±SEM. *P<0.05; **P<0.01; ***P<0.001. One‐way ANOVA with Tukey's post hoc test was used.

**Figure 5:**
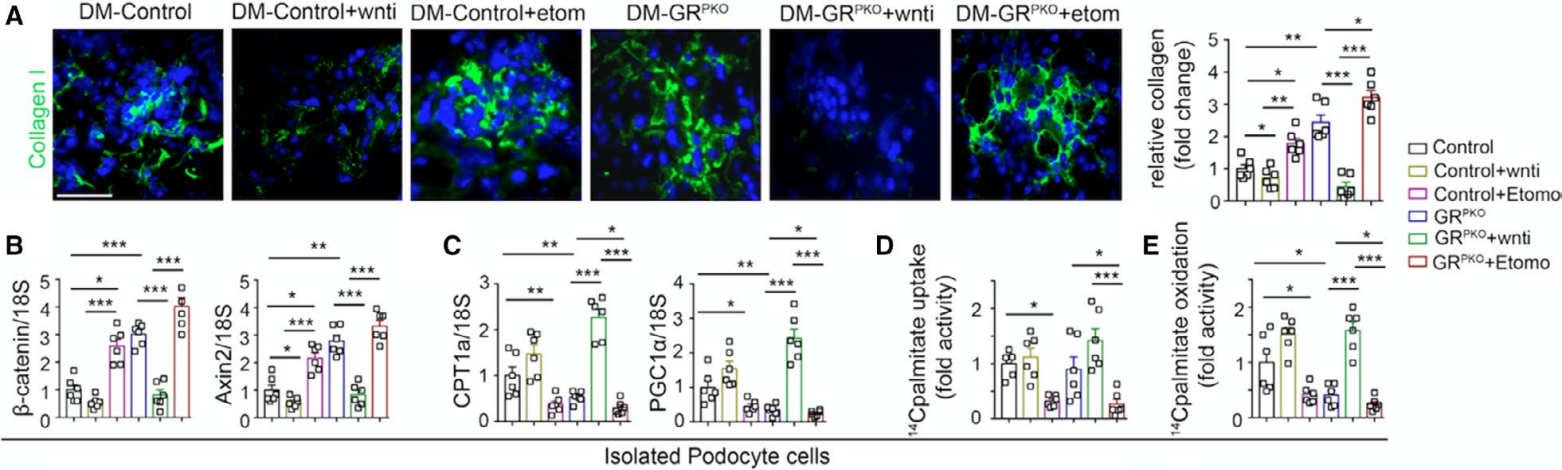
Wnt inhibitor increases fatty acid oxidation in podocytes. **A**, Immunofluorescence analysis and quantification of collagen I (fluorescein isothiocyanate–labeled collagen 1 and DAPI blue nuclei) in diabetic control and GR^PKO^ mice in the untreated, Wnti‐treated and etomoxir‐treated conditions. **B** and **C**, Quantitative polymerase chain reaction gene expression analysis of *β‐catenin*, *axin2*, *cpt1a*, and *pgc1α*, in podocytes isolated from mice subjected to the treatment conditions (**D**). Radiolabeled [C^14^]palmitate uptake analysis in isolated podocytes from indicated groups. CPM of each sample was counted. **E**, Radiolabeled [^14^C]palmitate oxidation and [^14^CO_2_] release were measured in indicated groups. Data mean±SEM. *P<0.05; **P<0.01; ***P<0.001. One‐way ANOVA with Tukey's post hoc test was used. CPM indicates counts per minute.

**Figure 6:**
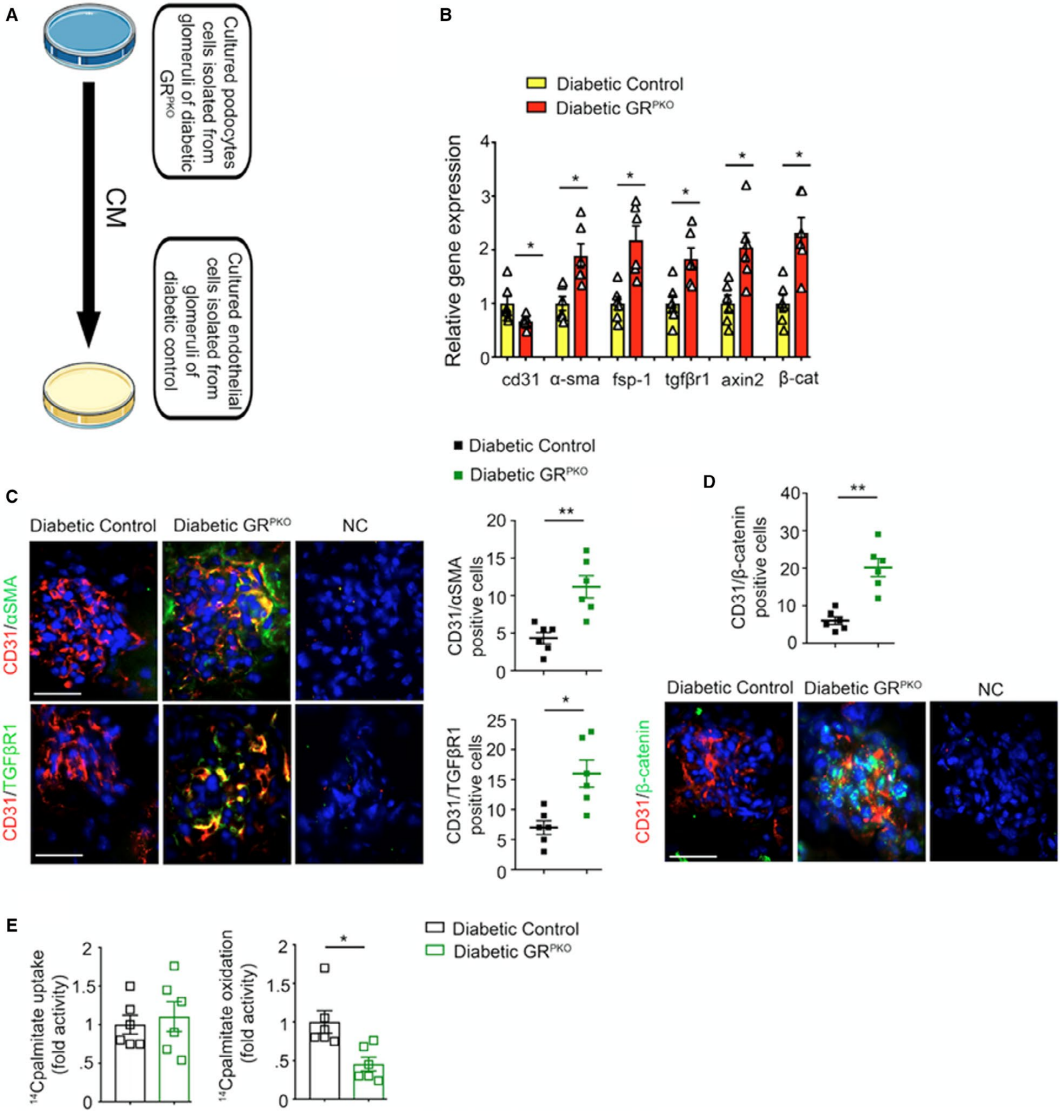
Glucocorticoid receptor loss in podocytes reprograms endothelial‐to‐mesenchymal transition processes in glomerular endothelial cells. **A**, Conditioned media (CM) experimental design. Isolated podocytes from the kidneys of diabetic podocyte‐specific glucocorticoid receptor knockout (GR^PKO^) and diabetic control mice were cultured for 96 hours. The subsequently harvested media was transferred to isolated endothelial cells from diabetic controls. **B**, Relative mRNA levels of endothelial‐to‐mesenchymal transition (EndMT) markers, canonical Wnt signaling genes and transforming growth factor (TGF) βR1 in conditioned media‐treated cultured endothelial cells from diabetic controls. **C**, Immunofluorescence analysis of /CD31αSMA, CD31/TGFβR1 and (**D**) CD31/β‐catenin was performed in the glomeruli of diabetic control and diabetic GR^PKO^ mice by fluorescence microscopy. (FITC‐labeled αSMA, TGFβR1 and β‐catenin; rhodamine‐labeled CD31 and DAPI nuclei blue). Representative images are shown. Scale bar: 50 μm in each panel (n=6/group). **E**, Radiolabeled [C^14^]palmitate uptake analysis in endothelial cells treated with conditioned media (CM) from indicated groups. CPM of each sample was counted. Radiolabeled [^14^C]palmitate oxidation was measured as indicated. Data are mean±SEM. *P<0.05, **P<0.01. NC indicates negative control. One‐way ANOVA with Tukey's post hoc test was used to analyze **B**; nonparametric Mann‐Whitney *U* test was used to analyze **C** through **E**. CPM indicates counts per minute.

Two‐way ANOVA was used in Figure 1B through 1h and the nonparametric Mann‐Whitney *U* test was used to analyze data in Figures 1I, 1J, 2, and 3. One‐way ANOVA with Tukey's post hoc test was used to analyze data in Figures 4, 5, and 6B. The nonparametric Mann‐Whitney *U* test was used to analyze Figure 6C through 6E. Data represent the number of separate experiments (in vitro) and the number of mice (in vivo). Technical replicates were used to ensure the reliability of single values. Data are expressed as mean±SEM. Statistical significance was accepted for *P*<0.05. Data were analyzed using the statistical package for Prism 7 (GraphPad Software, Inc., La Jolla, CA).

## RESULTS

### Loss of Podocyte GR Worsens DN

To investigate the effect of GR deletion in podocytes on the initiation and progression of diabetic nephropathy, we induced diabetes mellitus in control and GR^PKO^ mice with streptozotocin and monitored mice for 16 weeks (Figure 1A). Nondiabetic control and GR^PKO^ mice were also examined at the 16‐week time point. Diabetic animals had higher glucose, lower body weight, higher kidney and liver weight, lower heart weight, and higher plasma creatinine and albumin‐to‐creatinine ratios (ACRs) than nondiabetic animals, as expected (Figure 1B through 1H). Diabetic GR^PKO^ mice demonstrated higher plasma creatinine and ACR compared with diabetic control animals (Figure 1G and 1H).

Histologic analysis of kidneys isolated at 16 weeks post‐streptozotocin from diabetic control and GR^PKO^ mice showed increased glomerulomegaly and mesangial area in diabetic GR^PKO^ mice; however, there was no difference in interstitial fibrosis between the groups (Figure 1I and 1J). Immunohistochemistry for markers of fibrosis including TGFβ1 and p‐smad2 revealed increased expression in GR^PKO^ mice (Figure 2A). Immunofluorescent staining for collagen I, and TGFβ1/nephrin costaining in kidney sections from both genotypes also showed increased expression in diabetic GR^PKO^ mice compared with diabetic controls, indicating more severe glomerular fibrosis (Figure 2B).

The phenotype of these animals was also evaluated using an accelerated model of nephropathy, which combined both the streptozotocin‐induced diabetic model as well as the UUO model. In this model, control and GR^PKO^ mice were subjected to UUO followed by 4 daily doses of streptozotocin and euthanasia on day 12 (Figure S1A). There were no differences in body weight, blood glucose or kidney weight between the 2 groups but UUO‐operated GR^PKO^ mice demonstrated higher plasma creatinine and ACR compared with UUO‐operated control mice (Figure S1B). Histologic analysis of control and UUO‐operated kidneys from both genotypes demonstrated similar interstitial fibrosis but significantly greater glomerulomegaly and glomerular fibrosis in UUO‐operated kidneys from GR^PKO^ mice. There was no difference in fibrosis between the genotypes in the contralateral kidneys (Figure S1C). Immunohistochemistry for TGFβ1 and p‐smad2 again showed increased expression in GR^PKO^ mice (Figure S2A), as did immunostaining for collagen I, and TGFβ1/nephrin costaining (Figure S2B). These data suggest that the loss of podocyte glucocorticoid receptor leads to glomerular fibrosis in both diabetic conditions.

### Upregulation of Canonical Wnt Signaling and Metabolic Derangement in the Absence of Podocyte GR

To investigate whether Wnt signaling was altered in this mouse model, immunohistochemical staining for β‐catenin, the final effector molecule of the canonical Wnt signaling pathway, was performed in kidney sections from animals of both genotypes subjected to both renal fibrosis models. As shown in Figure 3A and Figure S3A, both diabetic GR^PKO^ mice and UUO‐operated GR^PKO^ mice had increased β‐catenin staining compared with their respective controls. Similarly, nephrin positive cells displayed higher β‐catenin staining in diabetic GR^PKO^ mice and UUO‐operated GR^PKO^ mice compared with controls (Figure 3B, Figure S3B), suggesting that GR loss‐linked activation of canonical Wnt signaling is a key driver of the development of diabetic kidney disease. To examine Wnt signaling specifically in podocytes, primary podocytes were isolated from animals of both genotypes as previously described,^36^ and subjected to quantitative polymerase chain reaction for several key Wnt‐dependent genes (*β‐catenin* and *axin2*) as well as the inflammatory cytokine interleukin‐6, collagen I, and the gene *Cpt1a*, a critical regulator of FAO. Podocytes isolated from diabetic GR^PKO^ mice demonstrated significantly higher expression of *collagen I*, *β‐catenin*, *axin2*, and *interlukin‐6* and significantly lower expression of *Cpt1a* compared with those from diabetic control mice (Figure 3C). Costaining of glomeruli for nephrin/Cpt1a confirmed decreased expression of Cpt1a in kidney sections from diabetic GR^PKO^ mice (Figure 3D) as well as in UUO‐operated GR^PKO^ mice compared with those from UUO‐operated control mice as well (Figure S3C). Podocytes isolated from diabetic GR^PKO^ mice did not exhibit significant alteration in [^14^C]palmitate (lipid uptake); however, they did show significantly suppressed [^14^C]palmitate oxidation (FAO) and cellular ATP levels compared with those from diabetic control mice (Figure 3E through 3G). Cumulatively, these data suggest that GR loss in podocytes results in worsened glomerular fibrosis via up regulation of Wnt signaling and activation of profibrotic signaling pathways.

### Wnt Inhibition Ameliorates and Suppression in FAO Worsens Glomerular Fibrosis

To determine if modulation of either Wnt signaling or FAO could alter the fibrotic phenotype in our mouse model of diabetic nephropathy, animals of both genotypes were treated with either the small molecular Wnt inhibitor LGK974^38^ from 16 to 24 weeks following streptozotocin (Figure 4A), or the FAO inhibitor etomoxir from 16 to 18 weeks following streptozotocin (Figure 4B). Histologic images of kidneys from both diabetic control and GR^PKO^ mice treated with each compound are shown in Figure 4C. In diabetic control animals, there was a trend toward improvement in renal fibrosis after LGK974 treatment, while clearly worsened fibrosis was observed after etomoxir treatment. In diabetic GR^PKO^ mice, LGK974 treatment significantly improved glomerular fibrosis and restored glomerular structure. Etomoxir had minimal effect in diabetic GR^PKO^ mice. These histologic results were closely approximated by the observed ACR for each group with Wnt inhibitor–treated animals demonstrating improved ACR compared with controls and etomoxir‐treated animals demonstrating worsened ACR (Figure 4D).

Immunofluorescence analysis of collagen I in each of the conditions tested showed that Wnt inhibition decreased overall collagen I expression, while etomoxir increased such expression (Figure 5A). Podocytes isolated from diabetic control animals subjected to Wnt inhibitor treatment showed a slight decrease in the expression of key Wnt‐dependent genes (*β‐catenin*, *axin2)* pro‐fibrotic genes (*collagen 1*, *fibronectin 1*, *tgfr1β*) and a small increase in the expression of genes in the FAO pathway (*cpt1a*, *pgc1α*). Podocytes isolated from diabetic control animals subjected to etomoxir treatment showed dramatically higher expression of Wnt‐dependent (*β‐catenin*, *axin2)* profibrotic genes (*collagen 1*, *fibronectin 1*, *tgfr1β*) and lower expression of genes in the FAO pathway (*cpt1a*, *pgc1α*) compared with diabetic control podocytes (Figure 5B and 5C; Figure S4). Podocytes isolated from diabetic GR^PKO^ animals subjected to Wnt inhibitor treatment showed a dramatic decrease in *β‐catenin*, *axin2*, *collagen 1*, *fibronectin 1*, and *tgfr1β*, while the expression of *cpt1a* and *pgc1α* was significantly upregulated. Conversely, podocytes isolated from diabetic GR^PKO^ animals treated with etomoxir demonstrated higher expression of *β‐catenin*, *axin2*, *collagen 1*, *fibronectin 1*, and *tgfr1β,* and lower expression of *cpt1a* and *pgc1α* compared with podocytes isolated from the diabetic GR^PKO^ mice (Figure 5B and 5C; Figure S4). Podocytes isolated from diabetic control and diabetic GR^PKO^ animals subjected to Wnt inhibitor treatment did not show any significant change in [^14^C]palmitate uptake when compared with their respective nontreated diabetic groups. However, Wnt inhibition dramatically increased the [^14^C]palmitate oxidation in diabetic GR^PKO^ animals when compared with untreated diabetic GR^PKO^ group. Podocytes isolated from diabetic control and diabetic GR^PKO^ animals subjected to etomoxir treatment demonstrated significant suppression in [^14^C]palmitate uptake and [^14^C]palmitate oxidation when compared with their respective untreated diabetic groups (Figure 5D and 5E).

### Loss of Podocyte GR Favors Endothelial‐to‐Mesenchymal Transition

To further investigate the anti‐fibrotic role of podocyte GR, we analyzed its interaction with glomerular endothelial cell metabolism and fibrosis. Fibrogenic processes in glomerular endothelial cells include endothelial‐to‐mesenchymal transition (EndMT) which is a key process that contributes to glomerular fibrosis in diabetic nephropathy.^13^, ^43^, ^44^, ^45^ Activated canonical Wnt signaling in endothelial cells can induce EndMT in glomeruli,^46^, ^47^ although neither endothelial cell–derived fibroblasts alone nor their intermediate cell types are sufficient to induce glomerular fibrosis.^46^, ^48^

To determine the mechanism by which loss of podocyte GR worsens glomerular fibrosis, primary podocytes from diabetic control and GR^PKO^ mice were isolated and cultured for 96 hours. The conditioned media were transferred to glomerular endothelial cells that had been isolated from diabetic control animals (Figure 6A) and also incubated for 96 hours. Endothelial cells treated with conditioned media from GR‐deplete podocytes showed increased mRNA expression of *α‐SMA*, *TGFβR1* and *β‐catenin*, *axin2* and *fsp1,* and decreased mRNA expression of *cd31* compared with endothelial cells treated with conditioned media from normal podocytes (Figure 6B). Immunofluorescent staining of isolated kidney sections from diabetic control and GR^PKO^ mice also demonstrated increased colocalization of these markers in endothelial cells (Figure 6C and 6D). Endothelial cells treated with conditioned media from GR‐depleted podocytes showed suppressed [^14^C]palmitate oxidation while [^14^C]palmitate uptake was unaltered (Figure 6E).

## Discussion

This study demonstrates the critical role of podocyte cell GR in the regulation of fibrogenic processes in the glomeruli in a mouse model of diabetic kidney disease. Our results demonstrate that podocyte GR regulates canonical Wnt signaling and fatty acid metabolism thereby affecting the mesenchymal transdifferentiation process in glomerular endothelial cells in diabetic mice. Podocyte GR loss is one of the fibrotic phenotypes in glomeruli. Consequently, these processes alter the metabolic switch in favor of defective fatty acid metabolism and its associated mesenchymal activation in glomerular endothelial cells, suggesting that GR loss in podocytes disrupts essential crosstalk between podocytes and endothelial cells.

Murine podocytes express GR^36^, ^49^ and, in response to treatment with dexamethasone, demonstrate GR phosphorylation, downregulation of GR protein, upregulation of GR‐responsive genes and translocation of GR to the nucleus.^49^ The effects of glucocorticoids on podocytes and the dependence of these effects have been studied in models both in vitro and in vivo.^50^ For example, dexamethasone has been shown to protect against puromycin aminonucleoside–induced injury by stabilizing the actin cytoskeletal network and increasing the activity of the guanosine triphosphatase RhoA.^51^ Dexamethasone has also been shown to protect podocytes from puromycin aminonucleoside–induced injury by induction of ERK phosphorylation and its associated anti‐apoptotic effect.^52^, ^53^

It is important to note that cortisol (or corticosterone in rodents) is the only endogenous ligand for GR, but tissue‐specific manipulation of GR can produce profound whole‐organism phenotypes that are not present when systemic alterations of glucocorticoid levels occur, as in Cushing disease. For example, deletion of GR in the central nervous system results in mice with profoundly altered hypothalamic‐pituitary‐adrenal axes and 10‐fold elevated circulating corticosterone levels as well as reduced anxiety‐related behavior.^54^ Tissue‐specific excision of GR from hepatocytes results in a severe growth deficit thought to be attributable to downregulation of STAT5‐mediated transcription.^55^ Mice with tissue‐specific deletion of GR in endothelial cells have been shown to be more susceptible to sepsis and atherosclerosis.^56^, ^57^ Available data also suggest that cellular responses to glucocorticoids will depend not only on the specific effector tissue but also the GR isoform composition, alternative splicing events, and posttranslational modifications.^58^ The complexity of the regulatory mechanisms of GR signaling underscores the importance of endogenous corticosterone in regulating normal homeostasis.

Our data suggest that GR deficiency in podocytes is a critical step in the metabolic reprogramming of the glomerular endothelium and contributes to DN.

Recently, we reported that loss of GR in endothelial cells results in upregulation of Wnt signaling and contributes to vascular inflammation in mice.^59^ Upregulation of Wnt signaling is recognized as a mechanistic underpinning of renal fibrosis.^60^ Enhanced Wnt signaling is linked with activation of profibrotic signaling, expression of inflammatory cytokines, and defective cell metabolism in other cell types in the kidney.^60^, ^61^, ^62^ Defective fatty acid metabolism can also contribute to the activation of profibrotic signaling in mouse models of diabetic kidney disease.^33^, ^34^ To date, most studies have examined the metabolism of tubular epithelial cells, endothelial cells, and mesangial cells; there is not yet a study that convincingly demonstrates the impact of podocyte metabolism on renal disease progression.^32^, ^63^, ^64^

Wnt inhibition in our model ameliorated the characteristics of glomerular segmental fibrosis in diabetic kidneys; it was further associated with restored fatty acid metabolism in podocytes. It is clear that inhibition of FAO in podocytes enhances profibrotic signaling and canonical Wnt signaling. These data suggest that Wnt inhibition improves glomerular fibrosis by augmenting FAO and suppressing the expression of profibrotic genes. The antifibrotic effect of Wnt inhibition appears to be dependent on podocyte GR, suggesting that GR is an essential protein that regulates podocyte Wnt signaling and its associated fatty acid metabolism in diabetes mellitus.

In addition, our results demonstrate that GR loss in podocytes induces mesenchymal activation in glomeruli suggesting crosstalk between podocytes and endothelial cells mediated via GR. There is at least 1 report that suggests that defects in podocytes can affect the permeability of the basement membrane,^1^ which could be a potential mechanism whereby endothelial cell homeostasis could be affected by podocyte derangements. Further studies will be required to investigate this mechanism more fully. In addition, similar to our results, aberrant activation of epithelial‐to‐mesenchymal transition in tubular epithelial cells is thought likely to induce EndMT processes and accelerate fibrotic events in diabetes mellitus.^65^

**Figure 7:**
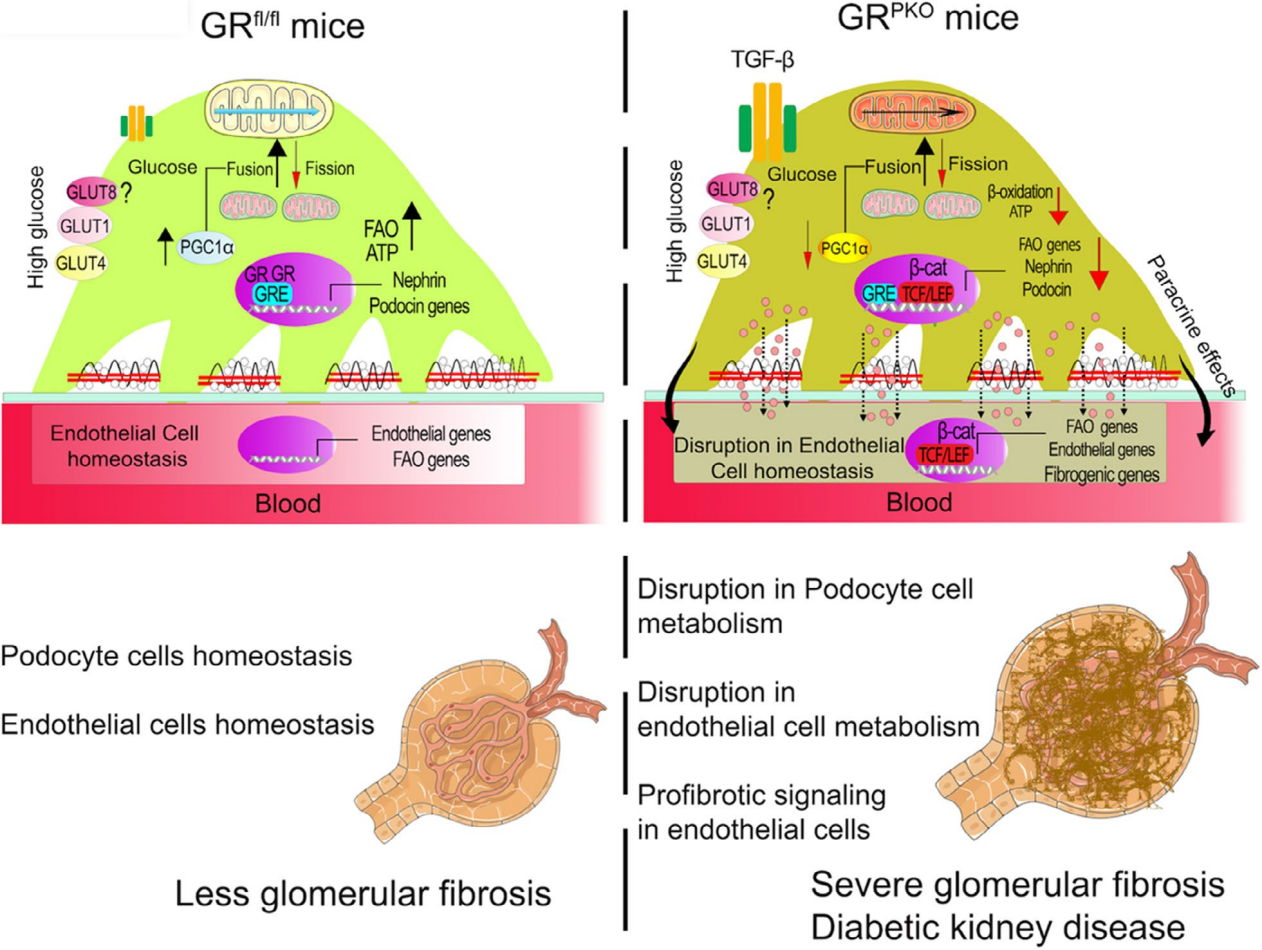
Working hypothesis. Podocyte glucocorticoid receptor loss causes metabolic shifts in glomerular endothelial cells and is a critical contributor to the fibrogenic response in diabetic glomeruli.

As outlined in Figure 7, the working hypothesis is that podocyte GR maintains homeostasis in both podocytes and the kidneys as a whole by maintaining normal cellular metabolism through FAO and relative suppression of Wnt signaling in podocytes. In the absence of podocyte GR, Wnt signaling is upregulated, FAO is suppressed, and EndMT is stimulated. We hypothesize paracrine effects from the absence of podocyte GR on endothelial cells catalyze the EndMT. There is yet no known ligand secreted by podocytes in the GR‐deficient state that definitively accounts for this observation, though the fact that endothelial cells treated with conditioned media from GR‐deficient podocytes have decreased FAO suggests that such a ligand exists. Our previous work in cell‐specific models of GR deficiency has shown that loss of endogenous steroid signaling in particular microenvironments, such as the endothelium, can predispose to inflammation, apoptosis, and fibrosis,^35^, ^56^, ^57^ thereby reinforcing the notion that endogenous steroid signaling has profound effects on cell homeostasis.

Our study has some interesting translational implications. Though steroids are generally not considered as a therapy for diabetes mellitus because of a predictable exacerbation in glycemic control, the ability to deliver steroids in a tissue‐specific manner, such as directly to podocytes, may hold promise as a novel therapeutic repurposing of steroid therapy. Indeed, this work has already begun in some animal models of glomerular disease. In a mouse model of lupus nephritis, subcutaneous delivery of liposome‐based steroidal nanodrug was shown to have superior efficacy compared with free (nonliposomal) glucocorticoid in terms of overall survival, kidney pathological injury score, and blood urea nitrogen concentrations.^66^ A liposomal encapsulation approach of prednisolone has also been used to study the response of human endothelial cells and macrophages subjected to lipopolysaccharide treatment. In this work, liposomal uptake by macrophages was complete 8 hours after administration, and cells showed decreased levels of the proinflammatory cytokine interleukin‐6 as well as evidence of nuclear translocation of GR, at least as efficaciously as free prednisolone alone.^67^ Such studies are slowly accumulating and form the basis for a growing argument to further develop cell‐specific steroid delivery technologies.^68^

In summary, our data demonstrate that GR deficiency in podocytes induces Wnt signaling that is responsible for defects in fatty acid metabolism. The cumulative effects of enhanced canonical Wnt signaling and defective fatty acid metabolism in podocytes lead to mesenchymal activation in glomerular endothelial cells and associated fibrogenesis in glomeruli. The present study adds valuable information to current knowledge about GR biology in podocytes and its effects on endothelial cell physiology in diabetes mellitus.

## Sources of Funding

This work is supported by grants from the National Institutes of Health to Drs Dardik (R01HL128406), Fernandez‐Hernando (R35HL135820), and Goodwin (R01HL131952).

## Disclosures

None.

## Supporting information

Figures S1–S4
