# Supplementary material for: Podocyte Glucocorticoid Receptors Are Essential for Glomerular Endothelial Cell Homeostasis in Diabetes Mellitus
Source: J Am Heart Assoc. 2021 Jul 26;10(15):e019437. doi: 10.1161/JAHA.120.019437 (PMC8475689; doi:10.1161/JAHA.120.019437)
Supplement: Supplementary file 1 — Figures S1–S4 [file JAH3-10-e019437-s001.pdf]

SUPPLEMENTAL MATERIAL

Figure S1. Loss of podocyte GR worsens glomerular fibrosis in a mouse model of urinary obstruction (UUO).

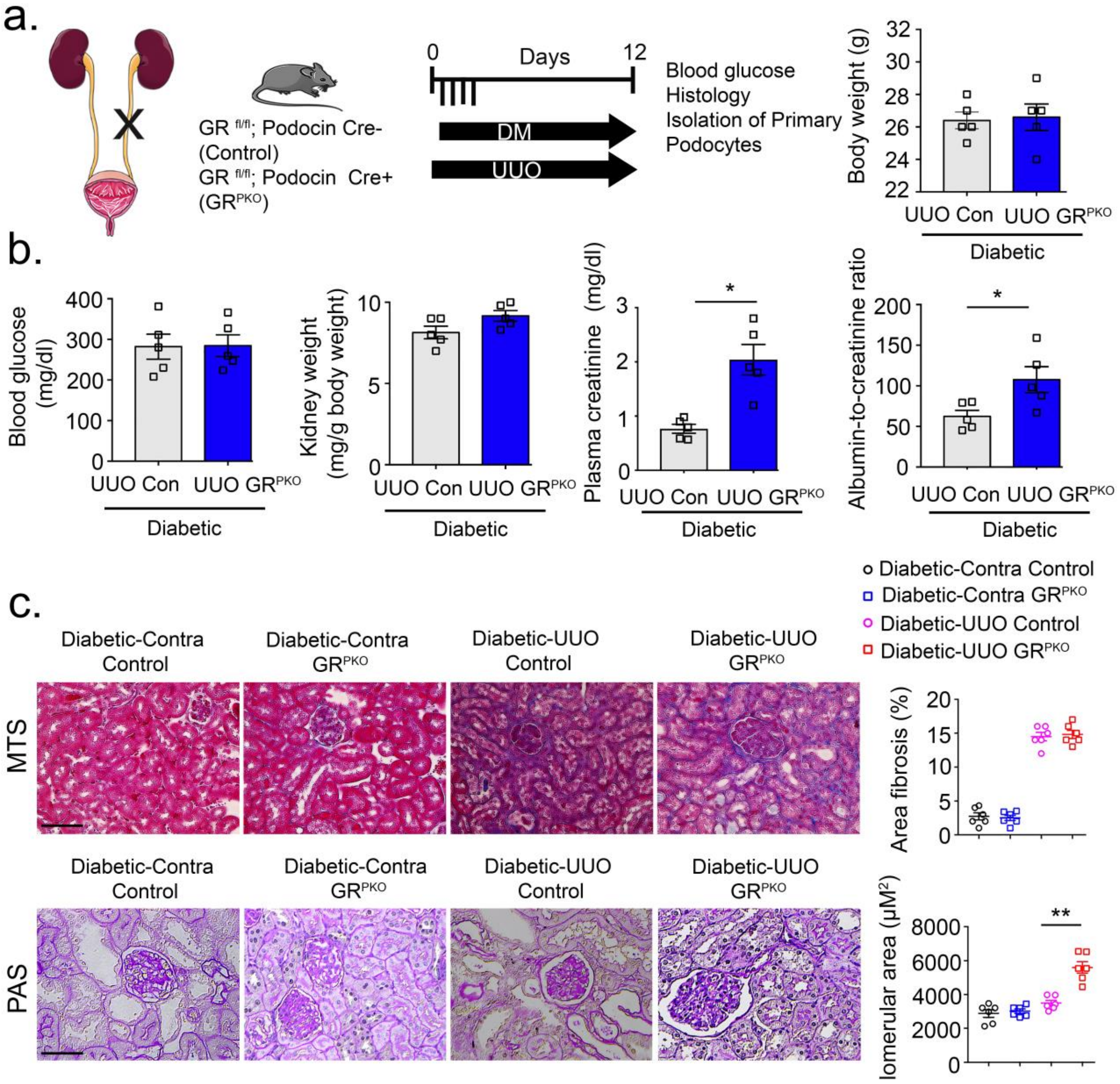

(a) Schematic presentation of UUO model. Left kidneys were ligated in control littermates and GR^{PKO} mice. Four doses of STZ (50 mg/kg/day, i.p.) were injected from day 2 post-UUO to induce diabetes. At day 12, kidneys were excised. **(b)** Physiological parameters: body weight, blood glucose, kidney weight/body weight, plasma creatinine and albumin-to-creatinine ratio were measured. n=5/group. Data are shown as mean ± SEM. **(c)** Masson trichrome (MTS) and

PAS staining in the contralateral and UUO-operated kidneys in control and GR^{PKO} mice were analyzed. Representative images are shown. Area fibrosis (%) and glomerular area (μM^2) were measured using ImageJ. n=6/group. Data are shown as mean ± SEM. Scale bar 50 μm. *$p<0.05$, **$p<0.01$. Non-parametric Mann-Whitney U test was used.

Figure S2. Loss of podocyte glucocorticoid receptor augments TGFβ-Smad2 signaling in UUO operated diabetic glomeruli.

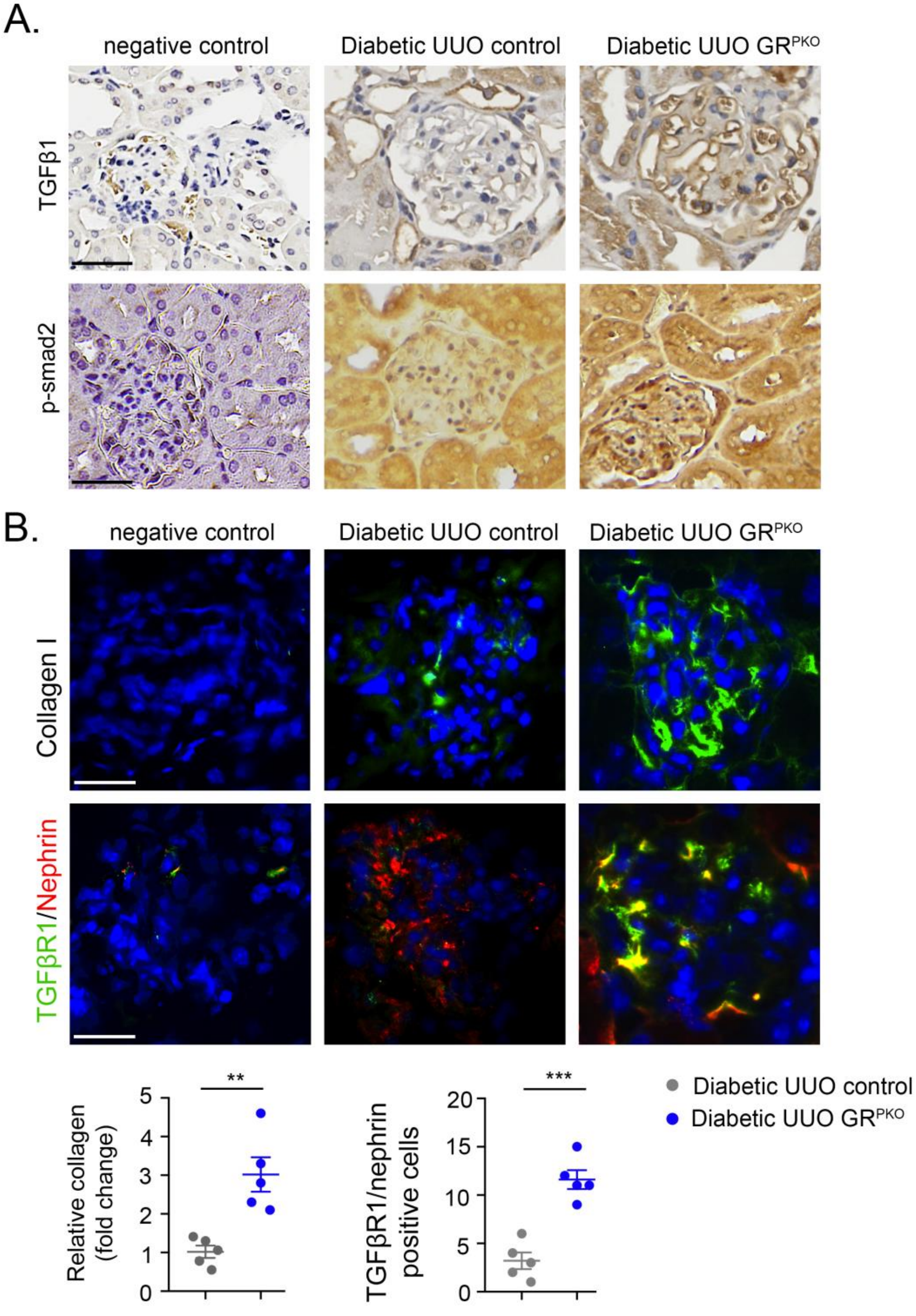

(a) Immunohistochemical analysis of TGFβ1and p-smad2 in the glomeruli of diabetic UUO control and diabetic UUO GR^{PKO} mice n=6/group. Representative pictures are shown. Scale bar 50 μm. **(b)** Immunofluorescence analysis and quantification of collagen I (FITC-labeled and DAPI blue nuclei, pixels/field) and TGFβR1and nephrin co-labeling (FITC-labeled TGFβR1, rhodamine-labeled nephrin and DAPI blue nuclei) in the glomeruli of diabetic UUO control and diabetic UUO GR^{PKO} mice. Representative images are shown. Scale bar: 50 μm in each panel. n=6/group. *$p<0.05$, **$p<0.01$. Non-parametric Manny-Whitney U test was used.

Figure S3. Aberrant Wnt signaling is responsible for podocyte GR loss-linked fibrosis in glomeruli of UUO-operated diabetic mice.

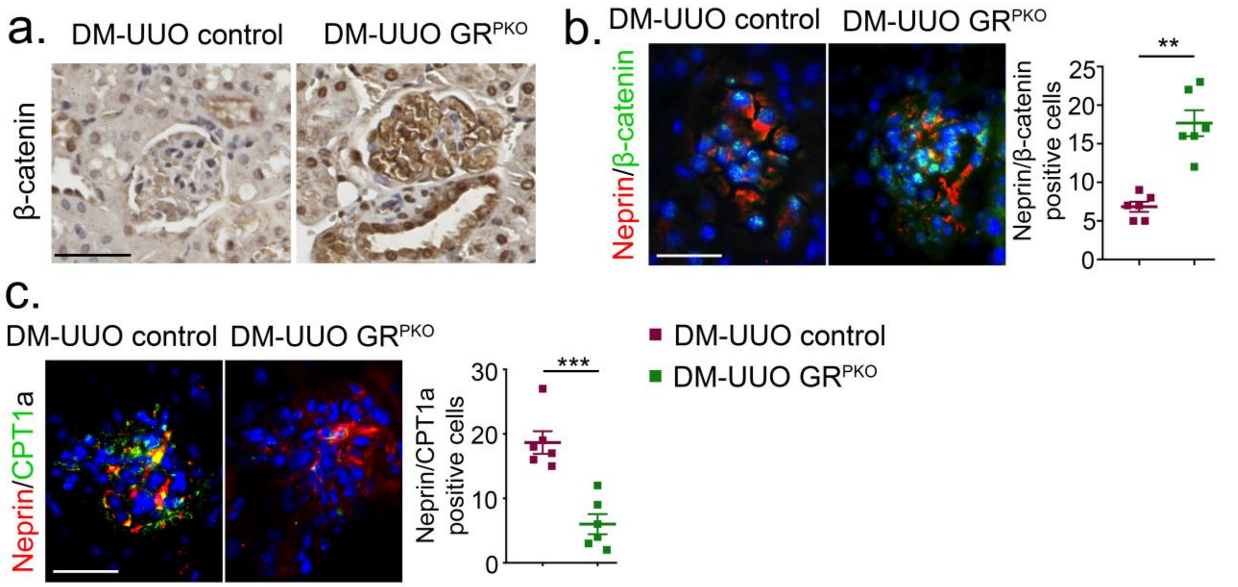

(a) Immunohistochemical analysis of β-catenin in the glomeruli of diabetic UUO control and diabetic UUO GR^{PKO} mice. n=5/group. Representative images are shown. Scale bar 50μm. **(b)** Immunofluorescence analysis of nephrin/β-catenin co-labeling in the glomeruli of diabetic UUO control and diabetic UUO GR^{PKO} mice. (FITC-labeled β-catenin, rhodamine-labeled nephrin and DAPI blue nuclei). Representative images are shown. Scale bar 50 μm in each panel. n=5/group. **(c)** Immunofluorescence analysis of nephrin/CPT1a co-labeling in the glomeruli of diabetic UUO control and diabetic UUO GR^{PKO} mice. (FITC-labeled CPT1a, rhodamine-labeled nephrin and DAPI blue nuclei). Representative images are shown. Scale bar 50 μm in each panel. n=5/group. **$p<0.01$, ***$p<0.001$. DM: diabetes mellitus. Non-parametric Mann-Whitney U test was used.

Figure S4. **Gene expression analysis of profibrotic genes in isolated podocytes.**

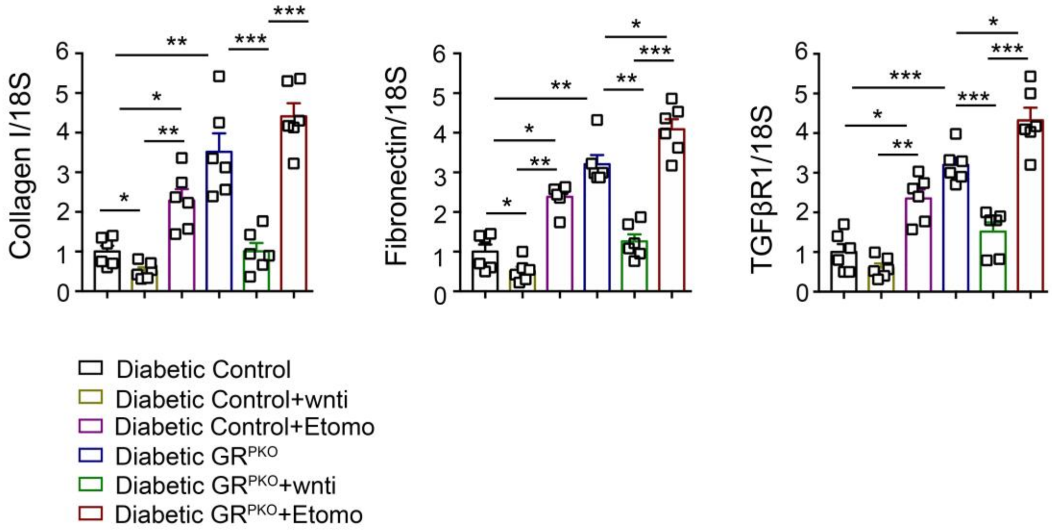

qPCR gene expression analysis of collagen I, fibronectin and TGFβR1 in podocytes isolated from mice subjected to the treatment conditions indicated. Data were normalized to 18S and are shown as mean ± SEM. *$p<0.05$, **$p<0.01$, ***$p<0.001$. One way ANOVA withTukey's post hoc test was used for analysis of statistical significance.
